# A Multi-Antenna Scheme for Early Detection and Mitigation of Intermediate GNSS Spoofing

**DOI:** 10.3390/s19102411

**Published:** 2019-05-27

**Authors:** Jaroslaw Magiera

**Affiliations:** Faculty of Electronics, Telecommunications and Informatics, Gdansk University of Technology, ul. G. Narutowicza 11/12, 80-233 Gdansk, Poland; jaroslaw.magiera@eti.pg.edu.pl

**Keywords:** GNSS spoofing, spoofing detection, spoofing mitigation, array processing, beamforming, multipath detection

## Abstract

This article presents a method for detecting and mitigating intermediate GNSS spoofing. In this type of attack, at its early stage, a spoofer transmits counterfeit signals which have slight time offsets compared to true signals arriving from satellites. The anti-spoofing method proposed in this article fuses antenna array processing techniques with a multipath detection algorithm. The latter is necessary to separate highly correlated true and counterfeit GNSS signals. Spoofing detection is based on comparison of steering vectors related to received spatial components. Whereas mitigation is achieved by means of adaptive beamforming which excises interferences arriving from common direction and preserves undistorted signals from GNSS satellites. Performance of proposed method is evaluated through simulations, results of which prove the usefulness of this method for protecting GNSS receivers from intermediate spoofing interference.

## 1. Introduction

Spoofing attacks are a well-known threat to the security of civilian GNSS receivers. This malicious interference resembles a bundle of genuine signals arriving from navigation satellites, yet with changed relative delays or carrying modified navigation messages. Spoofing is aimed to force target receiver to indicate incorrect position, velocity and time (PVT) information [1]. 

Due to low power of spoofing signals and their similarity to true signals, spoofing is far more difficult to detect than jamming. Numerous GNSS spoofing countermeasures have been proposed so far, varying in robustness and complexity [2,3,4]. Effectiveness of various spoofing detection algorithms depends on how sophisticated is the spoofing device (spoofer). Simplistic spoofing attacks may be conducted using an off-the-shelf GNSS constellation simulator connected to a power amplifier with a transmit antenna [5]. In this case target receiver is expected to lock to strong falsified signals while weaker genuine ones are jammed. This type of spoofing may be detected using relatively simple methods which usually require only modifications of receiver’s software. 

In more advanced spoofing scenario, called intermediate spoofing or carry-off attack, spoofer is directly connected with own GNSS receiver which provides current information about parameters of genuine signals [6]. It initially transmits signals which are precisely synchronized with signals arriving from navigation satellites to target receiver. Power of transmitted signals is gradually increased in order to take control over receiver’s tracking loops [7]. After that, relative delays between component signals are manipulated, resulting in calculated position drifting away from the true one. This kind of attack, if conducted properly, will be imperceptible to most of currently used civilian receivers. This means that, unlike in simplistic spoofing scenario, there will be no discontinuities in providing PVT information. Detection of such attack using methods restricted to receiver’s software is in most cases not effective. Multi-antenna signal processing is found to be the most robust approach among spoofing detection methods which do not employ cryptography nor require additional sensors such as inertial measurement units [8]. 

Multi-antenna receivers may still be spoofed in the case of coordinated transmission through a distributed network of antennas, when each component signal has a different direction of arrival (DOA) [6]. This scenario, called sophisticated spoofing, is however very unlikely to happen in practice since it is extremely difficult to conduct as long as receiver is not exposed to spoofing on purpose.

Various spatial processing methods for GNSS spoofing detection and mitigation have been proposed in the last several years. In [9,10] authors use a single moving antenna, which corresponds to synthetic antenna array, to analyze spatial patterns of received GNSS signals. Still, it is more often preferred to use a physical antenna array due to better performance and independence from antenna motion. In [11,12] dual-antenna carrier phase measurements are used for purpose of spoofing detection. In [13] an adaptive antenna array is used to both detect and mitigate spoofing basing on DOA analysis, incorporating receiver’s antenna attitude estimation. However, estimating actual DOA requires precise array calibration which may be cumbersome. In [14,15] the authors propose GNSS spoofing countermeasures based on analysis of signals’ steering vectors, assuming that these vectors may be estimated independently for authentic and spoofing signals. It may not be achieved if these signals are correlated as in case of early stage of intermediate spoofing.

In [16,17] our multi-antenna approach to detection and mitigation of GPS spoofing was presented. However, it was intended for scenarios where spoofing signals are significantly stronger than true signals and there is a relatively large time offset between them. This situation corresponds to either simplistic spoofing or late stage of intermediate spoofing. It is highly desirable that intermediate spoofing is mitigated before it introduces a significant error in position indicated by GNSS receiver. In this paper, a novel version of multi-antenna anti-spoofing scheme is presented, which allows to detect and filter out falsified GNSS signals at early stage of intermediate spoofing, i.e., in the beginning of carry-off phase. 

The remainder of this paper is organized as follows: the second part describes the mathematical model of GNSS signals in the presence of intermediate spoofing. Next, the proposed methods for spoofing detection and mitigation are described. The fourth part presents results of simulations which were conducted to validate the anti-spoofing algorithms. The final part presents the conclusions. 

## 2. Intermediate GNSS spoofing Signal Model

In normal conditions (i.e., without spoofing) *K_sat_* signals arriving from GNSS satellites are present at receiver’s antenna input. Baseband equivalent of a single signal incoming from *k*-th satellite may be written as:

(1) $$s_{sat}^{k}\left(t\right)=\sqrt{2P_{sat}^{k}}\times D^{k}\left(t-\tau_{sat}^{k}\right)\times C^{k}\left(t-\tau_{sat}^{k}\right)\times e^{i2\pi f_{D}^{k}\left(t-\tau_{sat}^{k}\right)},$$

where *D^k^* represents navigation data component, *C^k^*—pseudorandom (PRN) code component, $P_{sat}^{k}$—received power of *k*-th signal, $f_{D}^{k}$—carrier Doppler shift and $\tau_{sat}^{k}$—propagation delay from *k*-th satellite to receiver’s antenna phase centre. 

In case of spoofing attack, a bundle of *K_spoof_* GNSS signals is transmitted by spoofer. Each of spoofing signal’s components at receiver’s antenna input is described as:

(2) $$s_{spoof}^{k}\left(t\right)=\sqrt{2P_{spoof}^{k}}\times D^{k}\left(t-\tau_{sat}^{k}-\tau_{spoof}^{k}\right)\times C^{k}\left(t-\tau_{sat}^{k}-\tau_{spoof}^{k}\right)\;\times e^{i2\pi f_{D}^{k}\left(t-\tau_{sat}^{k}-\tau_{spoof}^{k}\right)}$$

where $\tau_{spoof}^{k}$ is an additional signal delay introduced by spoofer. It is assumed that spoofer has knowledge about receiver’s true position so it may calculate accurate $\tau_{sat}^{k}$ values basing on current GNSS ephemeris data. Moreover, spoofer disciplined by GPS clock can reproduce carrier Doppler shifts of genuine signal as observed by the target receiver.

**Figure 1:**
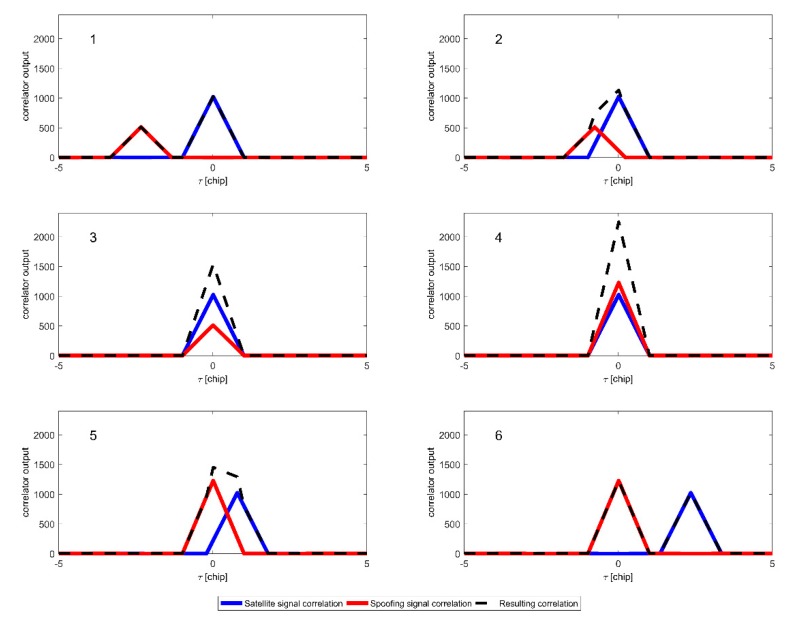
Correlator output during early phase of intermediate spoofing: code phase alignment (1–3), taking control over code tracking loops (4), carry-off—moving the correlation peak away from the true position (5,6).

In the first stage of intermediate spoofing, $\tau_{spoof}^{k}$ for each signal is set to zero, thus genuine and falsified signals arriving to receiver are time-aligned. After increasing $P_{spoof}^{k}$ to be greater than respective $P_{sat}^{k}$, $\tau_{spoof}^{k}$ values are modified to shift pseudorange measurements and, in consequence, affect indicated position information. The correlator output during consecutive carry-off spoofing phases is shown in Figure 1.

For a multi-antenna receiver, any component GNSS signal, either true or spoofing, in *m*-th receive chain is given by following formula:

(3) $$s_{sat\left(spoof\right)}^{m,k}\left(t\right)=s_{sat\left(spoof\right)}^{k}\left(t\right)\times a_{sat\left(spoof\right)}^{m,k}\times e^{i\phi_{m}}$$

where $a_{sat\left(spoof\right)}^{m,k}$ is a complex coefficient representing shift in signal’s amplitude and phase between *m*-th antenna element and array reference point. $\phi_{m}$ symbol represents signal phase shift introduced in *m*-th chain between receive antenna and ADC. This phase shift is equal for all received GNSS signals.

A set of all coefficients a of *k*-th signal received using *M*-element antenna array form a steering vector:

(4) $$a_{sat\left(spoof\right)}^{k}={\left[\begin{array}{cc} \begin{array}{cc} a_{sat\left(spoof\right)}^{1,k} & a_{sat\left(spoof\right)}^{2,k} \end{array} & \begin{array}{cc} \cdots & a_{sat\left(spoof\right)}^{M,k} \end{array} \end{array}\right]}^{T}$$

For a fixed antenna array geometry, the steering vector is related to the incoming signal’s direction-of-arrival. Thus, knowing that all falsified signals arrive from the same direction, it is possible to detect GNSS spoofing by comparing steering vectors of received signals. Evaluated values of common steering vector related to falsified signals are then used to control an adaptive spatial filter which excises the adverse spoofing component. Procedures for early detection and mitigation of intermediate GNSS spoofing are described in next section.

## 3. Early Detection and Mitigation of Spoofing

Assuming that each GNSS signal arrives to target receiver along a single path, composite signal received through *m*-th sensor of antenna array may be described as:

(5) $$s^{m}\left(t\right)=\overset{K_{sat}}{\underset{k=1}{\sum }}s_{sat}^{m,k}\left(t\right)+\overset{K_{spoof}}{\underset{k=1}{\sum }}s_{spoof}^{m,k}\left(t\right)+\eta_{m}\left(t\right)$$

where *η_m_*(*t*) represents additive noise which is uncorrelated between any pair of antennas.

**Figure 2:**
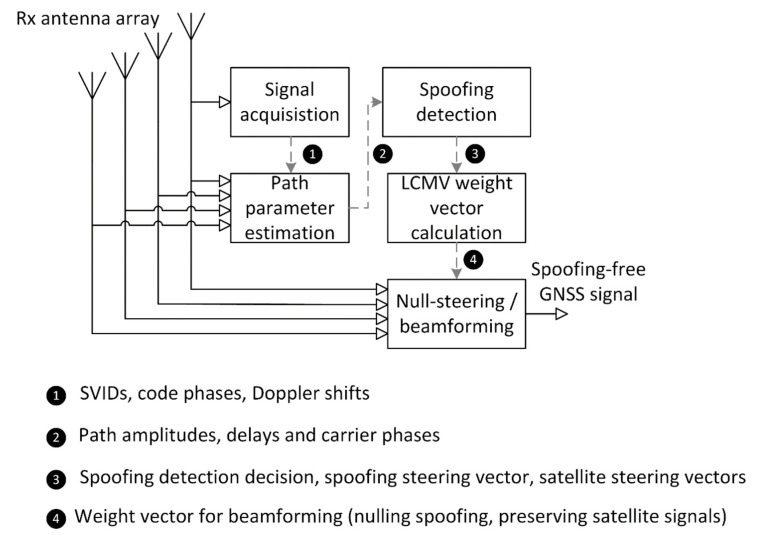
Scheme for detecting and mitigating intermediate GNSS spoofing.

In concept shown in Figure 2, signal (5) impinges on the receive antenna array. Output signals from all array sensors are shifted to lower frequency band and converted to digital form (these steps were not shown for brevity). Next, signal from a single sensor is processed in acquisition block which determines the indices of received GNSS signals, their carrier Doppler shifts and PRN code phase shifts. While signal acquisition is inherent to any GNSS receiver, remaining blocks in Figure 2 are specific to proposed anti-spoofing scheme. The procedure consists of three stages described in the following subsections.

### 3.1. Steering Vector Estimation

Prior to actual spoofing detection, individual steering vectors need to be estimated. Determining them requires all component signals in (5) to be separated. In absence of spoofing, particular signals may be extracted basing on their unique spreading codes (CDMA – GPS, Galileo) or occupied frequency channel (FDMA - GLONASS). However, when spoofing signals interfere with true signals, estimation of steering vectors is not straightforward. Both signals assigned to the same space vehicle ID (SVID) may be tracked separately if their relative delay is large enough so that they are represented as distinguishable peaks in correlation function. Minimum required delay is equal to one chip duration, which in case of GPS L1 signals is 1 microsecond that corresponds to 300 m of pseudorange difference. In early phase of intermediate spoofing, relative delays between genuine signals and respective falsified signals are much smaller. 

In order to detect signals with similar code delays, methods may be adopted which are already used for multipath detection in GNSS receivers. Majority of them is based on multiple correlator architecture, on successive multipath component cancellation or on maximum likelihood (ML) principle. In this article the last option was chosen as it employs optimum algorithm which provides most accurate parameter estimation. 

Considering reception period without 180-degree phase transitions related to navigation data, baseband equivalent of received multipath signal received through *m*-th antenna may be presented in the following way [18]:

(6) $$s^{m,k}\left(t\right)=\overset{L^{k}}{\underset{l=1}{\sum }}A_{l}^{m,k}\times C^{k}\left(t-\tau_{l}^{k}\right)\times e^{i\phi_{l}^{m,k}}+\eta^{m}\left(t\right)$$

assuming that the carrier Doppler shift is compensated in the receiver. $L^{k}$ symbol in (6) represents the number of multipath components in signal from the *k*-th satellite. Each component is characterized by parameters $A_{l}^{m.k}$, $\tau_{l}^{k}$ and $\phi_{l}^{m,k}$ representing its amplitude, delay and phase shift at *m*-th array sensor. In ML approach such values of these parameters are sought, which minimize loss function Γ defined as square error between received multipath signal $s^{m,k}$ and its estimate:

(7) $$\Gamma \left({\hat{A}}^{m,k},{\hat{\tau }}^{k},{\hat{\phi }}^{m,k}\right)=\int {\left|s^{m,k}\left(t\right)-\overset{L^{k}}{\underset{l=1}{\sum }}{\hat{A}}_{l}^{m,k}\times C^{k}\left(t-{\hat{\tau }}_{l}^{k}\right)\times e^{i{\hat{\phi }}_{l}^{m,k}}\right|}^{2}\;dt$$

where ${\hat{A}}^{m.k}$, ${\hat{\tau }}^{k}$, ${\hat{\phi }}^{m,k}$ represent $L^{k}$-element vectors of respective parameter estimates for *k*-th GNSS signal. Under assumption that each received signal has only two components, i.e., a true one and the spoofing one, Equation (7) may be written as:

(8) $$\Gamma \left({\hat{A}}^{m,k},{\hat{\tau }}^{k},{\hat{\phi }}^{m,k}\right)\;=\int {\left|s^{m,k}\left(t\right)-{\hat{A}}_{sat}^{m,k}\times C^{n}\left(t-{\hat{\tau }}_{sat}^{k}\right)\times e^{i{\hat{\phi }}_{sat}^{m,k}}-{\hat{A}}_{spoof}^{m,k}\times C^{k}\left(t-{\hat{\tau }}_{spoof}^{k}\right)\times e^{i{\hat{\phi }}_{spoof}^{m,k}}\right|}^{2}\;dt$$

The major disadvantage of the ML approach is its high computational complexity, which often prevents its practical application. In a multipath scenario, complexity grows exponentially with the number of spatial components. Even when there are only two spatial paths, ML procedure involves searching for optimal solution in a six-dimensional space $\left[{\hat{A}}_{1}^{m,k},\;{\hat{A}}_{2}^{m,k},{\hat{\tau }}_{1}^{k},{\hat{\tau }}_{2}^{k},{\hat{\phi }}_{1}^{m,k},{\hat{\phi }}_{2}^{m,k}\right]$, which is challenging for real-time implementation. However, in such case it is possible to apply an estimator called MMT (*Multipath Mitigation Technology*), which substantially reduces computational complexity [19]. The principle of MMT is to transform the vector of unknown parameters in such way that scanning is performed only in two-dimensional space, while optimal values of remaining four parameters are calculated using closed-form formulas. The loss function is decomposed to a sum of in-phase (I) and quadrature (Q) components. For *k*-th GNSS signal impinging on *m*-th element of antenna array it may be written as:

(9) $$\Gamma \left({\hat{A}}^{m,k},{\hat{\tau }}^{k},{\hat{\phi }}^{m,k}\right)=\int {\left[I^{m}\left(t\right)-a\times C^{k}\left(t-{\hat{\tau }}_{1}^{k}\right)-b\times C^{k}\left(t-{\hat{\tau }}_{2}^{k}\right)\right]}^{2}\;dt+\;\int {\left[Q^{m}\left(t\right)-c\times C^{k}\left(t-{\hat{\tau }}_{1}^{k}\right)-d\times C^{k}\left(t-{\hat{\tau }}_{2}^{k}\right)\right]}^{2}dt,$$

where $I^{m}$ and $Q^{m}\left(t\right)$ are real and imaginary parts of the overall signal received through the *m*-th antenna, and:

(10) $$a={\hat{A}}_{1}^{m,k}\times \cos\left({\hat{\phi }}_{1}^{m,k}\right),\;b={\hat{A}}_{2}^{m,k}\times \cos\left({\hat{\phi }}_{2}^{m,k}\right),\;c={\hat{A}}_{1}^{m,k}\times \sin\left({\hat{\phi }}_{1}^{m,k}\right),\;d={\hat{A}}_{2}^{m,k}\times \sin\left({\hat{\phi }}_{2}^{m,k}\right).$$

It should be noted that ${\hat{\tau }}^{k}$ is considered to be independent from *m*, since relative delays of signal propagation between array sensors introduce negligibly small shifts in code phase. On the other hand, amplitudes and carrier phase shifts for different antennas may vary significantly.

The optimum solution is sought within ${\hat{\tau }}^{k}=\left[{\hat{\tau }}_{1}^{k},{\hat{\tau }}_{2}^{k}\right]$ space, where, for each considered pair of code delays, values of parameters *a*, *b*, *c* and *d* are evaluated which locally minimize the loss function. These are obtained by solving following equations:

(11) $$\begin{array}{cc} \begin{array}{cc} \frac{\partial \Gamma }{\partial a}=0 & \frac{\partial \Gamma }{\partial b}=0 \end{array} & \begin{array}{cc} \frac{\partial \Gamma }{\partial c}=0 & \frac{\partial \Gamma }{\partial d}=0 \end{array} \end{array}$$

Once the global minimum of loss function is found, values of $\left[{\hat{A}}_{1}^{m,k},\;{\hat{A}}_{2}^{m,k},{\hat{\phi }}_{1}^{m,k},{\hat{\phi }}_{2}^{m,k}\right]$ may be calculated by solving set of equations (10). Next, for each spatial path, a steering vector is created in the following form:

(12) $$a_{p}^{k}={\left[\begin{array}{cc} \begin{array}{cc} {\hat{A}}_{p}^{1,k}\times e^{i{\hat{\phi }}_{p}^{1,k}} & {\hat{A}}_{p}^{2,k}\times e^{i{\hat{\phi }}_{p}^{2,k}} \end{array} & \begin{array}{cc} \cdots & {\hat{A}}_{p}^{M,k}\times e^{i{\hat{\phi }}_{p}^{M,k}} \end{array} \end{array}\right]}^{T}$$

### 3.2. Spoofing Detection

Respective steering vectors are related to signals’ directions of arrival, thus they may be used for detecting multiple signals originating from a single antenna. In proposed method, spoofing is detected when four or more GNSS signals arrive from the same direction. This assumption is based on the fact that at least four falsified signals are required to induce incorrect position. It is expected that in real spoofing scenarios the number of counterfeit signals will be similar to the number of visible satellites. Less than four spoofing signals could be possibly discarded by RAIM algorithm [20], as they would be considered inconsistent with majority of received genuine signals. 

In order to assess whether two spatial components arrive from the same direction, their steering vectors are compared. Since a spoofer may transmit signals with different amplitudes and carrier phase shifts, it is necessary to normalize steering vectors before comparing them. It is performed by simply dividing all components of vector by the value of its first component:

(13) $$\overset{˘}{a_{p}^{k}}=\frac{a_{p}^{k}}{{\hat{A}}_{p}^{1,k}\times e^{i{\hat{\phi }}_{p}^{1,k}}}$$

The distance between any two steering vectors is defined as a complex norm of their difference:

(14) $$\Delta a_{i,j}=\|a_{i}-a_{j}\|$$

**Figure 3:**
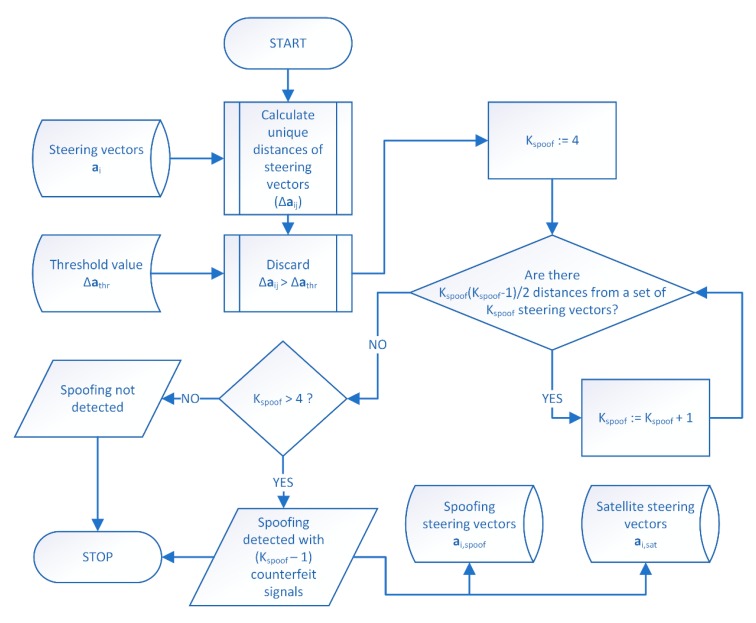
GNSS spoofing detection algorithm.

When steering vectors for all received spatial components are determined, spoofing detection is performed, following the algorithm presented in Figure 3.

First, for all pairs of steering vectors, their distances are calculated. For *K* steering vectors there are *K*(*K* − 1)/2 unique distances $\Delta a_{i,j}$. Next, distances are compared against the threshold value $\Delta a_{thr}$. The ones which are above this threshold are discarded as they are considered too large for signals arriving from the same direction. Initially, a hypothetical number of spoofing signals *K_spoof_* is set to four as a minimum number of signals required to estimate receiver’s position. Then, all possible *K_spoof_*-combinations of steering vectors are analyzed. For each combination of vectors it is checked whether all of their distances are included in the set of remaining $\Delta a_{i,j}$ (i.e., not greater than $\Delta a_{thr}$). If the test result is positive, *K_spoof_* is increased by one and the check is repeated. Otherwise, *K_spoof_* − 1 spoofing signals are detected if *K_spoof_* is greater than four. In other case, the algorithm ends without spoofing detection. 

In the case of positive spoofing detection, an alarm should be raised to warn the user that the indicated position or time information may not be correct. Apart from that, steering vectors are divided into two groups—one group contains steering vectors related to genuine signals, while the other one contains steering vectors related to counterfeit signals. This information is passed to spoofing mitigation block.

### 3.3. Determining Optimal Spoofing Detection Threshold

In the spoofing detection procedure, a threshold $\Delta a_{thr}$ is set which is used to distinguish pairs of spoofing steering vectors from other pairs of steering vectors. It is required to evaluate optimal threshold which will maximize the probability of spoofing detection with acceptably small probability of false alarm. A false alarm is considered as a situation when spoofing signals are not present, but detection is positive since for *K_sat_* signals received from satellites there are [*K_sat_* × (*K_sat_* − 1)/2] distances of steering vectors which do not exceed $\Delta a_{thr}$. It may occur when noisy steering vectors are evaluated for satellite signals which arrive from narrow spatial sector. 

Probability of spoofing detection may be estimated using the following formula:

(15) $$P_{d}=P\left[\underset{1\leq i<K}{\forall }\;\underset{j>i}{\forall }\left(\Delta a_{ij}\leq \Delta a_{thr}\right)\right]=\overset{K-1}{\underset{i=1}{\prod }}\overset{K}{\underset{j=i+1}{\prod }}\overset{\Delta a_{thr}}{\underset{0}{\int }}\left\{p\left(\Delta a_{ij}\right)\right\}d\Delta a_{ij}$$

where $p\left(\Delta a_{ij}\right)$ is the probability density function (PDF) of the distance between *i*-th and *j*-th steering vectors. When signals arrive from the same direction, as in spoofing case, $p\left(\Delta a_{ij}\right)$ depends only on *C/N*_0_ values of components related to particular SVs. On the other hand, when no-spoofing case is considered, $p\left(\Delta a_{ij}\right)$ is also a function of true non-zero distance between given pair of steering vectors. Thus, in order to evaluate the threshold value for which acceptable probability of false alarm is not exceeded, the worst case no-spoofing scenario should be analyzed with smallest nominal distances $\Delta a_{ij}$ observed in practice. When maximum threshold value is found, probability of spoofing detection may be evaluated using (15).

### 3.4. Spoofing Mitigation

Basically, spoofing is mitigated by means of adaptive control of the antenna reception pattern. When using an antenna array consisting of *M* sensors, *M*-1 degrees of freedom are available for steering spatial nulls or pointing beams towards particular directions. If a composite spoofing signal arrives from only one direction (or it has a dominant line-of-sight component), a single spatial null will suffice to remove this undesired component before it is passed to the GNSS receiver’s input. However, setting a single null without any other constraints may lead to a significant SNR decrease of desired signals from GNSS satellites as well. This is because a null exploiting only a single degree of freedom is spread over a range of directions which contain DOA of spoofing component, but may also include DOAs of genuine signals. It is possible to make use of all available degrees of freedom to establish a deep and spatially narrow null. However, in such case it is still not possible to control the distortion of signals from GNSS satellites. The better approach is to use each of remaining *M*-2 degrees of freedom to put a constraint setting 0 dB gain for given genuine signal. Minimum Variance Distortionless Response (MVDR) is a beamforming scheme which minimizes the output power while preserving a component arriving from desired direction [21]. Here, a modified version of MVDR is used, called Linearly Constrained Minimum Variance (LCMV) which allows for more than one constraint [22]. In this case, one constraint places a null for undesired spoofing components while other ones assure undistorted reception of signals from satellites. It should be noted that the main optimization criterion for both MVDR and LCMV beamforming algorithms is minimizing the power at the output of the antenna array. This means that, apart from spoofing mitigation, strong interference such as jamming will be removed as well, as long as its DOA does not coincide with DOA of acquired satellite signal. 

In practice, due to noise and mutual interference between GNSS signals, steering vectors estimated for every spoofing component are not exactly the same. Because of that, it is recommended to place a spatial null for each spoofing steering vector instead of a single null which could provide insufficient mitigation performance.

Furthermore, at most *M-K_spoof_* − 1 steering vectors of true GNSS signals are selected. These signals will be protected from distortion caused by array processing. 

A steering matrix **A** is created, composed of steering vectors as its columns. The first *K_spoof_* columns are the steering vectors of spoofing components, while remaining columns are steering vectors of genuine signals’ components:

(16) $$A=\left[\begin{array}{cc} \begin{array}{cc} \begin{array}{ccc} {\overset{˘}{a}}_{spoof}^{1} & \cdots & {\overset{˘}{a}}_{spoof}^{K_{sppof}} \end{array} & {\overset{˘}{a}}_{sat}^{1} \end{array} & \begin{array}{cc} \cdots & {\overset{˘}{a}}_{sat}^{M-K_{sppof}-1} \end{array} \end{array}\right]$$

If number of satellite steering vectors is smaller than *M-K_spoof_* − 1, number of columns in matrix **A** is reduced respectively.

Next, constraints vector **g** is created in the following form:

(17) $$g={\left[\begin{array}{cc} \underset{Kspoof}{\underbrace{\begin{array}{ccc} 0 & \cdots & 0 \end{array}}} & \underset{M-Kspoof-1}{\underbrace{\begin{array}{ccc} 1 & \cdots & 1 \end{array}}} \end{array}\right]}^{T}$$

where zeros correspond to the spatial null constraints and ones represent the non-distortion constraints. Number of elements in vector **g** is the same as the number of columns in matrix **A**.

Signal *y* at the output of the adaptive antenna array is a linear combination of array inputs forming vector **x** multiplied by weight coefficients forming a weight vector **w**.

(18) $$y\left[n\right]=w^{H}x\left[n\right]$$

where *n* is the index of time instant and *^H^* represents the Hermitian transpose. Weight vector for LCMV beamforming must fulfil the following equation:

(19) $$A^{H}w=g$$

There may be many solutions of Equation (19), but there is only one which also minimizes the output power. It is calculated using Equation (20):

(20) $$w_{LCMV}={\hat{R}}_{xx}^{-1}A{\left(A^{H}{\hat{R}}_{xx}^{-1}A\right)}^{-1}g$$

where ${\hat{R}}_{xx}$ is the array input covariance matrix estimate based on *N* snapshots:

(21) $${\hat{R}}_{xx}=\frac{1}{N}\overset{N}{\underset{n=1}{\sum }}x\left[n\right]x^{H}\left[n\right]$$

Output from the LCMV beamformer, being a digital waveform without components caused by spoofing or jamming, may be reconstructed in the analog domain and upconverted to its original frequency band. In such form it may be passed to RF input of GNSS receiver which is supposed to be protected from intentional interference.

When neither spoofing nor jamming are present, weight vector corresponding to single antenna reception should be applied:

(22) $$w={\left[\begin{array}{cc} \begin{array}{cc} 1 & 0 \end{array} & \begin{array}{cc} \cdots & 0 \end{array} \end{array}\right]}^{T}$$

Another possibility is to use a g vector with all ones, when number of array sensors is at least one more than number of visible satellites.

## 4. Simulation Results

Performance of the proposed anti-spoofing method was evaluated through computer simulations. These include: a) evaluation of optimum threshold for steering vector distances and b) verification of spoofing detection and mitigation in two spoofing scenarios.

**Figure 4:**
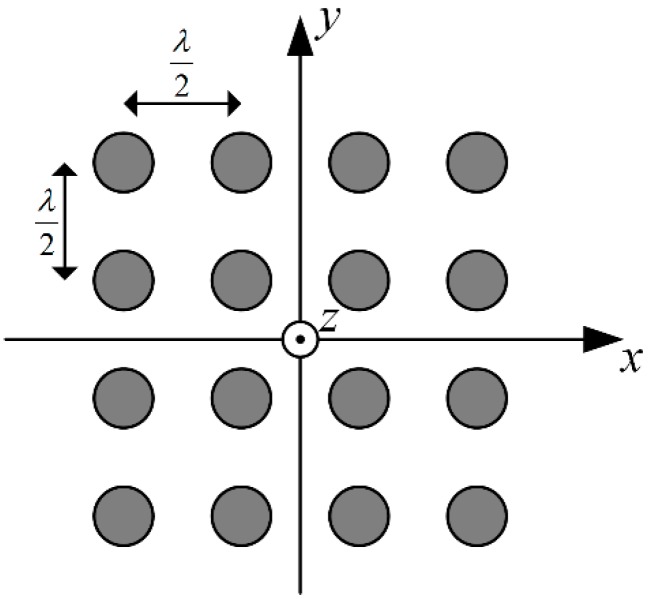
Arrangement of sensors in simulated model of antenna array.

Signal processing procedure was implemented in accordance with scheme presented in Figure 2. Ten-millisecond waveforms of signals received through array sensors were generated, following the formula (5). GPS L1 C/A signal structure was used, but it should be noted that presented approach generally applies to all GNSS signals based on spread spectrum technique. Additive noise components were uncorrelated between array sensors. a uniform rectangular antenna array (URA) model was applied, consisting of sixteen sensors arranged as shown in Figure 4. The XY plane is assumed to be parallel to the ground with Z axis pointing upwards. The distance between closest sensors is equal to one half of the wavelength at L1 carrier frequency.

The MMT algorithm was applied to estimate parameters of spatial components. Delay of the first path was sought in range ± 0.5 microseconds around the correlation peak obtained from acquisition stage. This search span corresponds to duration of a single chip in GPS C/A code, whereas the relative delay of the second path from the first path was sought in range from 0 to 100 nanoseconds. 

### 4.1. Evaluation of Detection Threshold

It is assumed that probability of false alarm *P_fa_* should not exceed 10^-2^. The worst-case scenario, where *P_fa_* is highest, is when four genuine signals arrive form similar directions resulting in small distances between steering vectors. In order to determine such conditions, GPS satellite trajectories were analyzed within 24-h period with 1-min step. Steering vectors were calculated corresponding to azimuth and elevation angles of signals arriving to a fixed location at 54° N latitude. It was found that for four signals arriving from directions [146°, 35°], [143°, 19°], [150°, 5°], [126°, 59°] a set of smallest true distances between steering vector is obtained, $\Delta a_{ij}\in$ {0.87, 0.95, 1.31, 2.54, 3.15, 3.67}. PDFs of $\Delta a_{ij}$ were estimated for integer values of C/N_0_ in range from 45 dBHz to 55 dBHz. For each value, Monte Carlo trials were conducted, assuming that C/N_0_ was equal for each of arriving spatial components. Empirical PDFs were derived from histograms of 1000 simulated output values. Using formula (15), maximum threshold values were estimated for which *P_fa_* < 10^−2^. Results are shown in Table 1. As may be seen, thresholds are lower for smaller C/N_0_ values because then PDFs are spread over a range of larger values of $\Delta a_{ij}$. According to Table 1, threshold value of 3.6 should be used in spoofing detection procedure as it constrains *P_fa_* the for all C/N_0_ values in the analyzed range.

**Figure 5:**
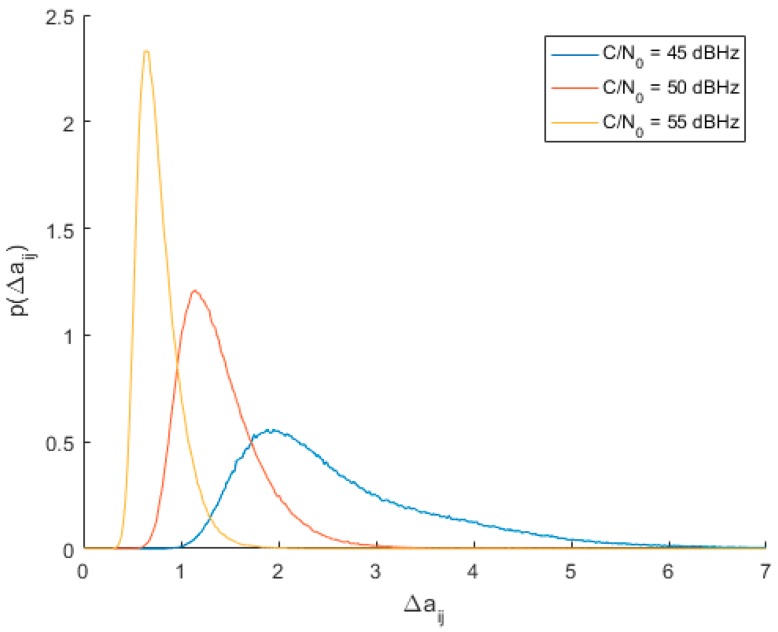
Empirical PDFs of spoofing steering vector distance for different C/N_0_ values.

Once the optimum threshold value was found, next simulations were conducted to evaluate the probability of spoofing detection. Another series of 1000 Monte Carlo trials were conducted to estimate the PDFs of $\Delta a_{ij}$ for signals arriving from the same direction with the same C/N_0_ in range from 45 to 55 dBHz. PDFs obtained for C/N_0_ values of 45, 50 and 55 dBHz are shown in Figure 5. 

Estimated probabilities of spoofing detection, calculated using Equation (15), are given in Table 2. As may be seen, best performance is observed for C/N_0_ not smaller than 50 dBHz, but even for smaller values *P_d_* is much greater than *P_fa_*.

### 4.2. Spoofing Scenario 1

In the first scenario six GPS satellite signals were received and each of these signals had a spoofing counterpart. All spoofing components arrived from direction [*ϕ_spoof_*,*θ_spoof_*] = {50°, 20°}, where *ϕ* and *θ* represent azimuth angle and elevation angle respectively. DOAs of genuine signals and their C/N_0_ values are given in Table 3.

**Figure 6:**
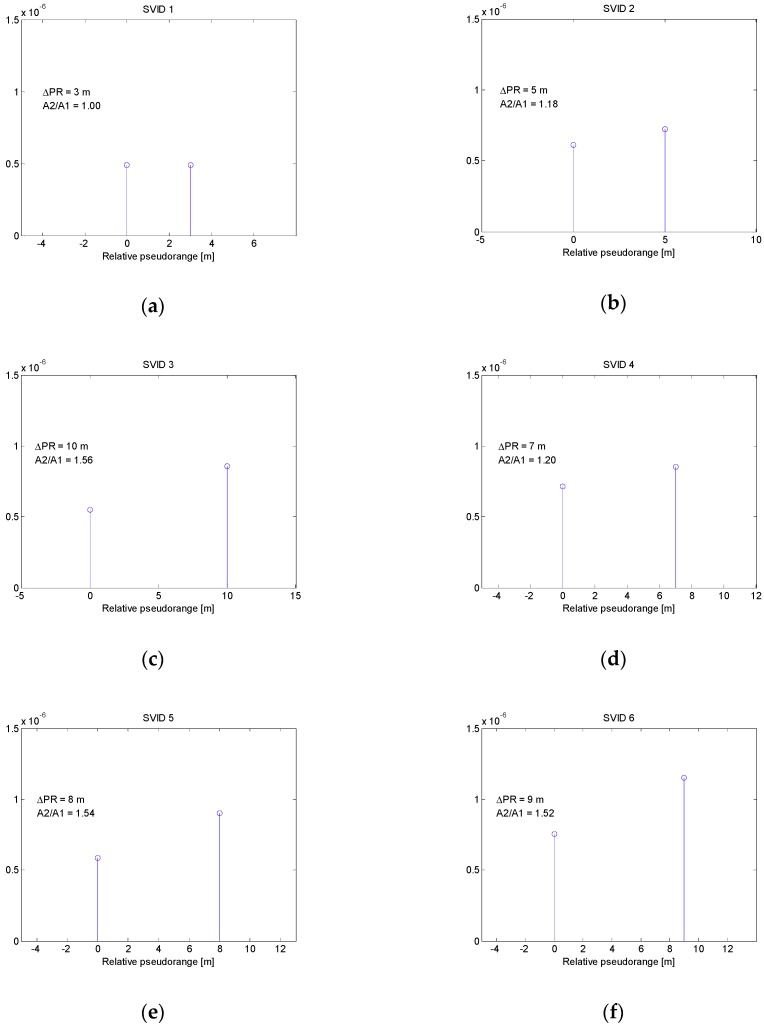
Multipath bins estimated in spoofing scenario 1 for signals related to SVIDs 1-6 (**a**–**f**).

Each signal from GPS satellite had a different code phase and Doppler shift. Simulated spoofing signals arriving to receiver were the delayed copies of true signals with twice greater power (3 dB higher C/N_0_). Introduced delays did not exceed 50 nanoseconds. It corresponds to the early stage of carry-off spoofing phase with relative pseudorange differences not greater than 15 m. In this scenario the actual pseudorange differences between counterfeit and true signals were set to: 3 m, 5 m, 10 m, 7, 8 and 9 m for SVIDs 1 to 6 respectively. Figure 6 presents the results of multipath detection procedure using MMT algorithm. For each SVID, estimated multipath bins are shown along with their relative pseudorange *ΔPR* and amplitude ratio *A2*/*A1*. All pseudorange differences between multipath bins were estimated correctly, while amplitude ratios varied from the true value of 1.41 (3 dB power ratio).

**Figure 7:**
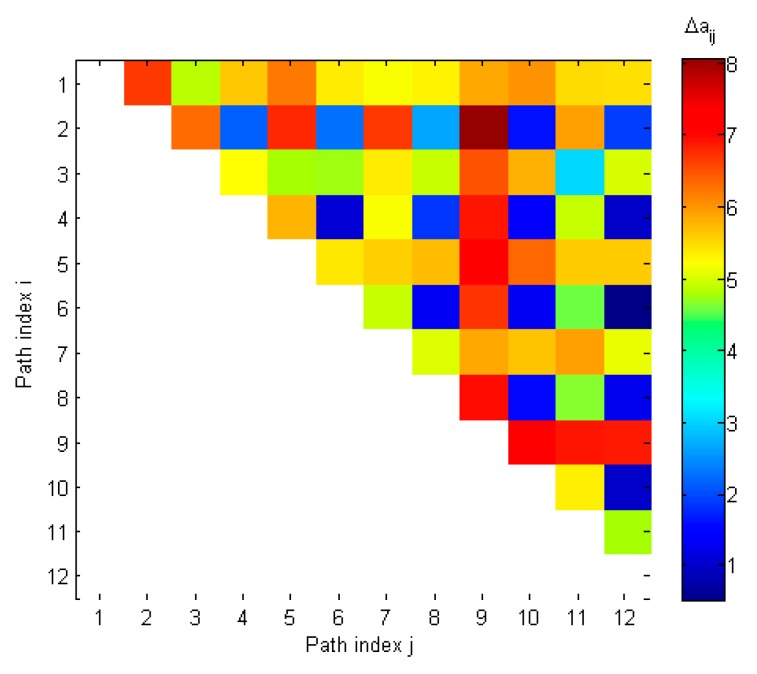
Distances between steering vectors in spoofing scenario 1.

Table 4 and Figure 7 present values of distances between steering vectors of all 12 detected spatial components. As may be seen from Table 4, there were 16 distances which were smaller than $\Delta a_{thr}$ = 3.6 (highlighted cells). Assuming that *K_spoof_* is equal to 6, the spoofing detection checks if there are 15 distances related to 6 components. It is visible that there was a set of 15 distances related to components 2, 4, 6, 8, 10 and 12. In result, spoofing was detected and counterfeit signals were identified as even spatial components, which is correct.

Two six-element sets of steering vectors of true and counterfeit signals were passed to weight calculation block, where optimal weight vector was determined using LCMV method. 

**Figure 8:**
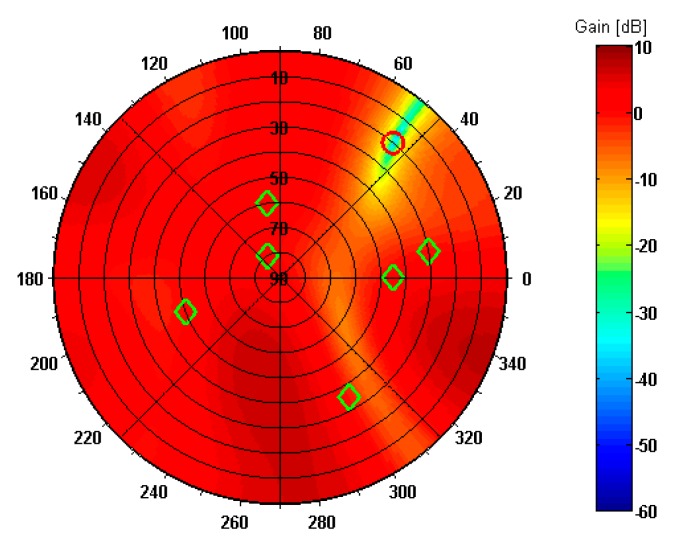
Reception pattern of antenna array in spoofing scenario 1.

The resulting pattern of antenna array with optimum set of weights applied is shown in Figure 8. The green diamond symbols indicate the DOAs of true signals according to Table 3, while the red circle symbol marks the DOA of composite spoofing signal. As may be seen, there is a deep null (over −30 dB) including direction of spoofing interference while preserving unity gains (0 dB) towards GPS satellites.

### 4.3. Spoofing Scenario 2

This simulation was conducted to prove the effectiveness of proposed method when the number of spoofing components is not equal to the number of visible satellites. In addition, the DOA of the spoofing signal was set to coincide with the DOA of one of satellite signals. These conditions may represent the situation where a spoofer is located on an aircraft, helicopter, unmanned aerial vehicle or on a high-altitude platform.

In this scenario the number of satellite signals, their DOAs and C/N_0_ values were the same as shown in Table 3. On the other hand, there were only four spoofing components imitating satellites with SVIDs from 1 to 4. These components were arriving from direction [*ϕ_spoof_*,*θ_spoof_*] = {100°, 60°}, which is the same as DOA of satellite with SVID 4.

**Figure 9:**
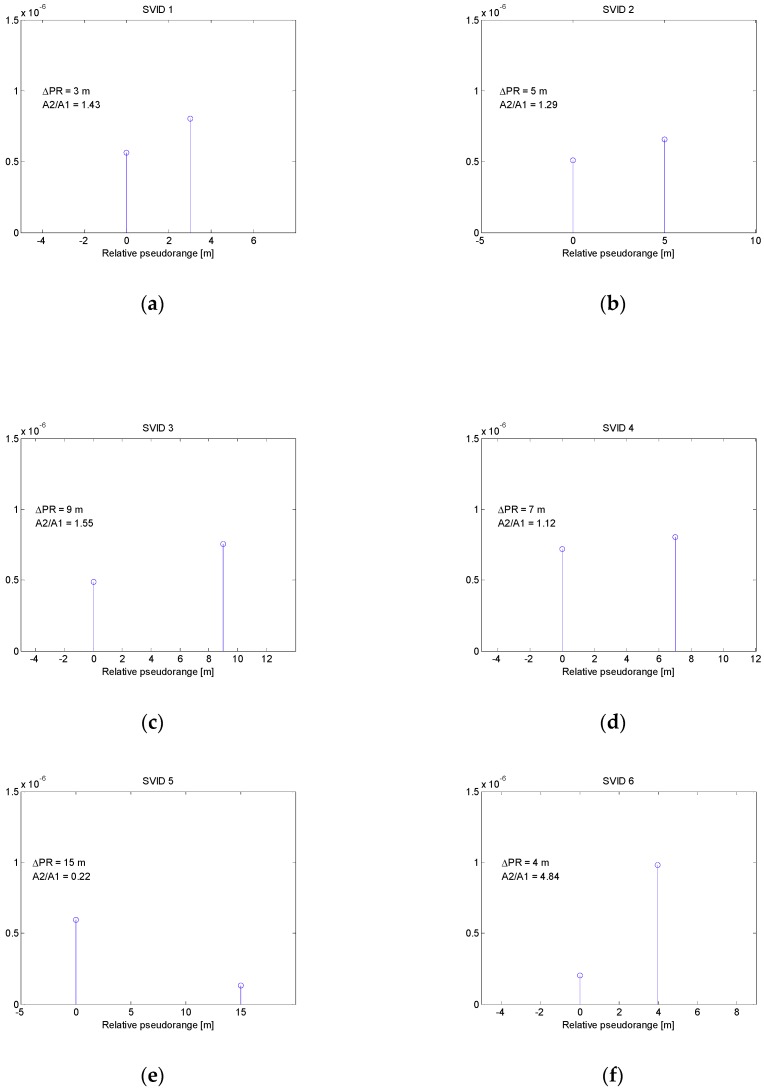
Multipath bins estimated in spoofing scenario 2 for signals related to SVIDs 1-6 (**a**–**f**).

Again, the MMT method was used to detect the spatial components. The results are presented in Figure 9. For SVIDs from 1 to 4 estimated bins are similar to the ones shown in Figure 6, except for SVID 3, where relative pseudorange Δ*PR* of spoofing component was found to be 9 m instead of actual 10 m. Also, estimated amplitude ratios do not differ significantly from the true value of 1.41. The results for SVIDs 5 and 6 expose one dominant bin as expected. Even if only a single spatial component is present, MMT returns two bins where one of them has much smaller amplitude than the other one.

**Figure 10:**
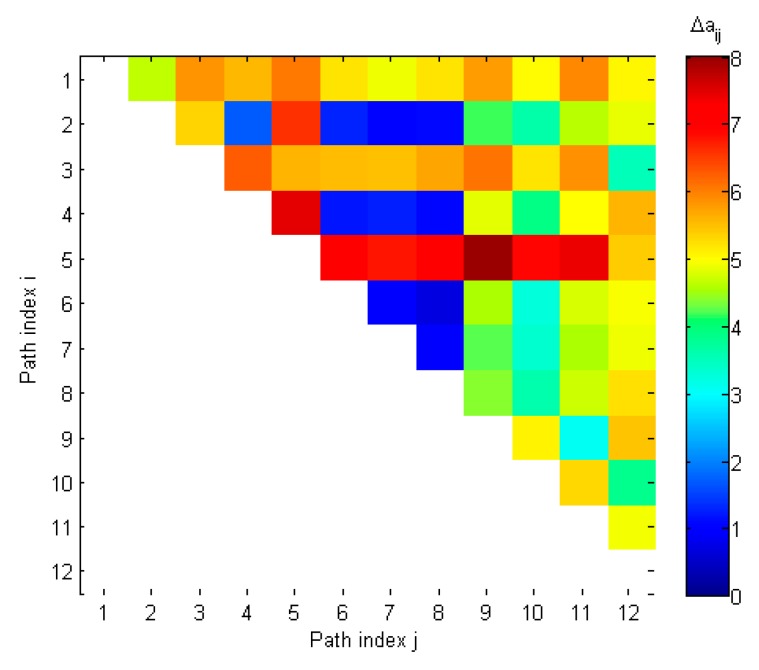
Distances between steering vectors in spoofing scenario 2.

During the spoofing detection procedure, assuming that *K_spoof_* is equal to 5, then 10 distances between steering vectors are selected. From Figure 10 and Table 5 it may be seen that there are fourteen distances smaller than $\Delta a_{thr}$. However, it is visible that a full set of steering vector distances occurs for the combination of components 2, 4, 6, 8 and 7, i.e., four spoofing components and one true signal.

In this case, one of true signals from GPS satellites is incorrectly classified as spoofing signal. However, since proposed anti-spoofing method is based solely on analysis of signals’ spatial signatures, it is not possible to decide which signal with SVID 4 is the true one and which is counterfeit.

**Figure 11:**
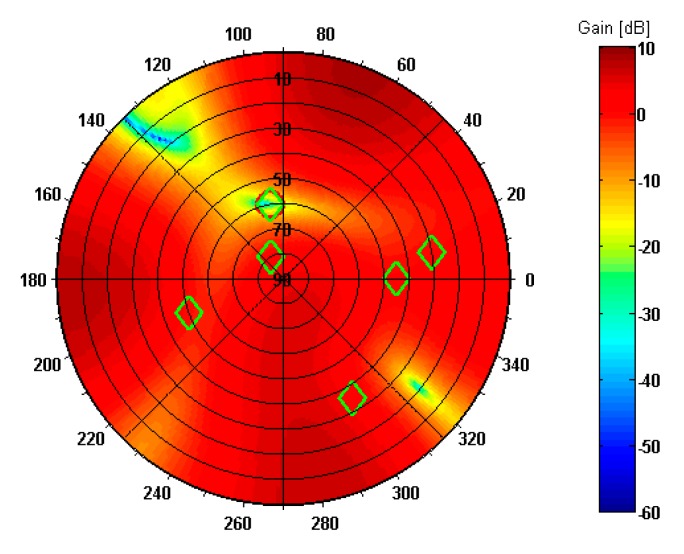
Reception pattern of antenna array in spoofing scenario 2.

The antenna array reception pattern evaluated for spoofing scenario 2 is presented in Figure 11. It is visible that a spatial null is formed in common DOA of spoofing components and one of true signals. For DOAs of other five true signals, unity gain is provided, as in scenario 1. This means that in this case spoofing is successfully mitigated at the cost of not receiving one of the true signals. If signals from at least four satellites remain, it will still be possible for the GPS receiver to estimate the 3D position.

## 5. Conclusions

The shown simulation results above prove the usefulness of the proposed method of detecting and mitigating intermediate GNSS spoofing at its early stage, which is a major advantage over other multi-antenna anti-spoofing solutions presented so far. Previously proposed methods could be still applied when spoofing signals are offset by more than one chip from respective true signals. Then it is not necessary to apply multipath detection technique, since these components may be acquired separately as their correlation peaks do not overlap.

Another asset is that proposed method combines spoofing detection and mitigation. When compared to [13,14], exploiting all spoofing and satellite steering vectors in LCMV beamforming provides greater control over antenna array pattern. Specifically, all spoofing components are nulled independently while genuine signals are preserved undistorted.

It should be also noted that described spoofing detection and spoofing mitigation methods both rely on knowledge about signals’ steering vectors. This is a substantial advantage over methods based on actual DOAs, like [13], as it eliminates the necessity to maintain local oscillator phase coherence between receive chains. This means that no phase calibration is necessary before using this anti-spoofing system, i.e., phase shifts $\phi_{m}$ in (3) may have arbitrary values. Still, it is required to maintain time and frequency synchronization between receive chains to avoid time-varying steering vectors.

Before described method may be applied in practice, it should be verified that antenna array may be located in a multipath-free environment. When the signal from a GNSS satellite or signals from a spoofer travel along more than one spatial channel, the MMT algorithm is not suitable for estimating steering vectors. In such case, other multipath detection methods need to be used, which cannot be based on Maximum Likelihood due to its high computational burden. An example of iterative GNSS multipath mitigation technique is described in [23].

Regarding computational complexity, the most computationally intensive stages of proposed algorithm are the signal acquisition and multipath estimation which are both related to spoofing detection. For a PC with a dual-core CPU running MatLab it took about one second to perform the MMT procedure for a single SVID (a pair of true and spoofing components). It is expected that this time could be significantly reduced if a DSP or FPGA hardware platform was used. Nevertheless, spoofing detection is considered to be not time-critical and may be performed periodically. On the other hand, spoofing mitigation must be done in real-time. It is possible to achieve this, as beamforming spatial filter is basically a linear combiner which exhibits low computational complexity (*M* complex multiplications and *M* − 1 additions).

Another practical aspect is the optimal number of elements in the antenna array. There is an inherent tradeoff between hardware/DSP complexity and effectiveness. More antennas may improve spoofing detection when C/N_0_ is low. Moreover, the number of sensors determines the degrees of freedom for controlling the array reception pattern. Recommended minimum number of antennas is one more than the total number of expected spoofing components and genuine signals from visible satellites. 

When spoofing is aimed at GNSS receivers installed in aerial vehicles, it is expected that the spoofer will be located higher than the antenna of the target. Such conditions were assumed in two scenarios described in Section 4. It was proven that spoofing may be mitigated even when counterfeit signals arrive from the same direction as one of the true signals.

## Figures and Tables

**Table 1:** Maximum Δ**a** threshold satisfying condition *P_fa_* < 10^−2^.

C/N_0_	Δa_thr,max_	C/N_0_	Δa_thr,max_
45	3.6	51	3.7
46	3.6	52	3.8
47	3.6	53	3.8
48	3.6	54	4.0
49	3.6	55	4.0
50	3.6		

**Table 2:** Probability of spoofing detection with $\Delta a_{thr}$ = 3.6.

C/N_0_	*P_d_*	C/N_0_	*P_d_*
45	46.9%	51	99.9%
46	57.9%	52	> 99.9%
47	74.4%	53	> 99.9%
48	83.5%	54	> 99.9%
49	97.2%	55	> 99.9%
50	99.2%		

**Table 3:** Parameters of simulated genuine GPS signals.

SVID	ϕ_sat_ [°]	θ_sat_ [°]	C/N_0_ [dBHz]
1	300	35	47
2	0	45	48
3	200	50	49
4	100	60	50
5	120	80	51
6	10	30	52

**Table 4:** Distances between steering vectors in spoofing scenario 1.

i,j	2	3	4	5	6	7	8	9	10	11	12
**1**	6.62	4.83	5.60	6.19	5.35	5.16	5.29	5.83	5.99	5.45	5.42
**2**		6.27	2.14	6.76	2.26	6.64	2.64	8.03	1.62	5.89	1.92
**3**			5.18	4.75	4.71	5.35	4.89	6.46	5.77	3.01	5.00
**4**				5.75	1.10	5.17	1.83	6.85	1.38	4.90	1.01
**5**					5.37	5.55	5.69	7.17	6.31	5.58	5.58
**6**						4.90	1.31	6.67	1.31	4.55	0.51
**7**							5.02	5.85	5.65	5.90	5.08
**8**								6.94	1.55	4.63	1.27
**9**									7.33	6.88	6.83
**10**										5.30	1.01
**11**											4.74

**Table 5:** Distances between steering vectors in spoofing scenario 2.

i,j	2	3	4	5	6	7	8	9	10	11	12
**1**	4.64	5.82	5.54	6.05	5.19	4.88	5.20	5.76	5.00	5.91	5.05
**2**		5.34	1.69	6.58	1.30	1.01	1.11	4.18	3.64	4.61	4.86
**3**			6.25	5.57	5.52	5.49	5.72	6.06	5.20	5.85	3.52
**4**				7.45	1.21	1.26	1.07	4.82	3.90	4.99	5.58
**5**					7.09	6.77	7.20	7.97	6.90	7.39	5.39
**6**						1.00	0.70	4.54	3.26	4.77	4.96
**7**							0.98	4.22	3.37	4.56	4.89
**8**								4.40	3.62	4.69	5.22
**9**									5.08	3.07	5.46
**10**										5.29	3.82
**11**											4.92

## References

[1] Ioannides R.T., Pany T., Gibbons G. (2016). Known Vulnerabilities of Global Navigation Satellite Systems, Status, and Potential Mitigation Techniques. Proc. IEEE.

[2] Warner J.S., Johnston R.G. (2003). GPS spoofing countermeasures. Homeland Secur. J..

[3] Jafarnia-Jahromi A., Broumandan A., Nielsen J., Lachapelle G. (2012). GPS Vulnerability to Spoofing Threats and a Review of Antispoofing Techniques. Int. J. Navig. Obs..

[4] Wen H.Q., Huang P.Y.-R., Dyer J., Archinal A., Fagan J. Countermeasures for GPS Signal Spoofing. Proceedings of the 18th International Technical Meeting of the Satellite Division of the Institute of Navigation (ION GNSS 2005).

[5] Dampf J., Pany T., Bär W., Winkel J., Mervart L., Ávila-Rodríguez J., Ioannides R., Hein G. (2017). Real World Spoofing Trials and Mitigation. InsideGNSS.

[6] Humphreys T.E., Ledvina B.M., Psiaki M.L., O’Hanlon B.W., Kintner P.M. Assessing the Spoofing Threat: Development of a Portable GPS Civilian Spoofer. Proceedings of the 21st International Technical Meeting of the Satellite Division of The Institute of Navigation (ION GNSS 2008).

[7] Psiaki M.L., Humphreys T.E. (2016). GNSS Spoofing and Detection. Proc. IEEE.

[8] Huang J., Lo Presti L., Motella B., Pini M. (2016). GNSS spoofing detection: Theoretical analysis and performance of the Ratio Test metric in open sky. ICT Express.

[9] Nielsen J., Broumandan A., Lachapelle G. (2010). Spoofing Detection and Mitigation with a Moving Handheld Receiver. GPS World.

[10] Psiaki M.L., Powell S.P., O’Hanlon B.W. GNSS Spoofing Detection using High-Frequency Antenna Motion and Carrier-Phase Data. Proceedings of the 26th International Technical Meeting of the Satellite Division of The Institute of Navigation (ION GNSS+ 2013).

[11] Psiaki M.L., OHanlon B.W., Powell S.P., Bhatti J.A., Wesson K.D., Humphreys T.E., Schofield A. GNSS Spoofing Detection Using Two-Antenna Differential Carrier Phase. Proceedings of the 27th International Technical Meeting of the Satellite Division of The Institute of Navigation (ION GNSS+ 2014).

[12] Borio D., Gioia C. (2016). A sum-of-squares approach to GNSS spoofing detection. IEEE Trans. Aerosp. Electron. Syst..

[13] Konovaltsev A., Caizzone S., Cuntz M., Meurer M. Autonomous Spoofing Detection and Mitigation with a Miniaturized Adaptive Antenna Array. Proceedings of the 27th International Technical Meeting of the Satellite Division of The Institute of Navigation (ION GNSS+ 2014).

[14] Daneshmand S., Jafarnia-Jahromi A., Broumandan A., Lachapelle G. A Low-Complexity GPS Anti-Spoofing Method Using a Multi-Antenna Array. Proceedings of the 25th International Technical Meeting of the Satellite Division of The Institute of Navigation (ION GNSS 2012).

[15] Daneshmand S., Jafarnia-Jahromi A., Broumandan A., Lachapelle G. A GNSS structural interference mitigation technique using antenna array processing. Proceedings of the 2014 IEEE 8th Sensor Array and Multichannel Signal Processing Workshop (SAM).

[16] Magiera J., Katulski R. Accuracy of differential phase delay estimation for GPS spoofing detection. Proceedings of the 36th International Conference on Telecommunications and Signal Processing (TSP).

[17] Magiera J., Katulski R. Applicability of Null-Steering for Spoofing Mitigation in Civilian GPS. Proceedings of the IEEE 79th Vehicular Technology Conference (VTC Spring).

[18] Sahmoudi M., Landry R. (2008). Multipath Mitigation Techniques using Maximum-Likelihood Principle. InsideGNSS.

[19] Weill L.R. Multipath Mitigation using Modernized GPS Signals: How Good Can it Get?. Proceedings of the 15th International Technical Meeting of the Satellite Division of the Institute of Navigation (ION GPS 2002).

[20] Parkinson B.W., Axelrad P. (1988). Autonomous GPS integrity monitoring using the pseudorange residual. Navigation.

[21] Capon J. (1969). High-resolution frequency-wavenumber spectrum analysis. Proc. IEEE.

[22] Frost O.L. (1972). An algorithm for linearly constrained adaptive array processing. Proc. IEEE.

[23] Chen X., Dovis F., Peng S., Morton Y. (2013). Comparative Studies of GPS Multipath Mitigation Methods Performance. IEEE Trans. Aerosp. Electron. Syst..

